# Internet Technology to Promote Academic Writing among Students and Trainees

**DOI:** 10.31662/jmaj.2023-0108

**Published:** 2023-09-29

**Authors:** Soichiro Saeki

**Affiliations:** 1 Center Hospital of the National Center for Global Health and Medicine, Tokyo, Japan

**Keywords:** Medical Education, Case Report, Letter to the Editor, Undergraduate, Postgraduate

I express my gratitude to Kakisaka et al. ^(1)^ for their keen interest in the preceding manuscript ^(2)^. I also found their curriculum on imparting writing skills to medical students through the implementation of internet technologies and the provision of motivation to publish intriguing.

The use of modern technologies such as YouTube and Google Docs, as demonstrated by the authors ^(1)^, presents various advantages. The younger population, particularly students and trainees, demonstrates expertise in using such technologies most frequently. Notably, the COVID-19 pandemic saw the emergence of remote learning ^(3)^. Harnessing such platforms would allow a broad educational outreach for various students and trainees. Second, by utilizing internet technology, it becomes possible to continue such educational programs irrespective of time and location, thereby facilitating uninterrupted utilization by young physicians who frequently undergo rotations across various clinical residency training facilities. Additionally, the authors’ online courses are conducted in Japanese ^(1)^, which successfully lowers the barriers for many students and trainees.

Furthermore, as the authors emphasize ^(1)^, fostering and sustaining motivation for writing is paramount in nurturing future researchers. To maintain such motivation, it is essential to engage in continuous writing, encompassing not only original articles but also concise manuscripts such as letters and case reports, which demand comparatively less effort. To the end, it is encouraged that editors of academic journals proactively consider accepting submissions from young researchers ^(2)^. Additionally, comprehensive educational programs within medical schools can motivate students through the influence of witnessing their peers’ publication achievements, thereby encouraging others to take initial steps. Such consistent efforts would ultimately establish solid groundwork for the culmination of research and contribution to the scientific academic community.

Finally, for students and trainees to become independent writers, education in medical ethics becomes imperative. Familiarity with obligations of confidentiality, informed consent, and when appropriate, adherence to an institutional review board are indispensable when handling patient information ^(4)^. It would be prudent to offer guidance on these matters during the early stages of education to motivated students and trainees.

As students and trainees, we have struggled through the COVID-19 pandemic. We missed out on our lifetime experiences ^(5)^ and were isolated from our peers. Nevertheless, we have persisted and adjusted by using new technological advancements and communication methods. Writing papers is not an easy task, but as the authors mention ^(1)^, writing can now be supported. It is imperative that we seize this opportunity to progress and overcome any potential challenges that lie ahead for the Japanese medical community.

## Article Information

### Conflicts of Interest

None

### Acknowledgement

The author is grateful to the author’s colleagues for helpful discussions on this topic. The author is also thankful to Paperpal (Cactus Communications Pvt. Ltd., Mumbai, India) and ChatGPT (OpenAI, L.L.C., San Francisco, CA, USA) for providing primary language editing. The views expressed in this manuscript are those of the author and do not necessarily represent those of the author’s institutions.

### Author Contributions

The author is solely responsible for the contents of the manuscript. Artificial intelligence technology was used for the language editing process only, and the author reviewed the contents after the language editing process. The author’s institution played no role in the conceptualization of this manuscript.

### Approval by Institutional Review Board (IRB)

Not applicable.
